# Sociodemographic and Clinical Characteristics of Functional Constipation in Children Receiving Care at a Tertiary Hospital: An Observational Study

**DOI:** 10.31729/jnma.v63i2091.9229

**Published:** 2025-11-30

**Authors:** Nirjala Aryal, Moon Thapa, Bishwo Raj Bahadur Kunwar, Bhumika G.C., Kusum Lamichhane

**Affiliations:** 1 Department of Paediatric Medicine, Shree Birendra Hospital, Chhauni, Kathmandu, Nepal; 2 Kathmandu University School of Medical Sciences, Dhulikhel, Kavrepalanchok, Nepal

**Keywords:** *constipation*, *prevalence*, *gastrointestinal disorder*, *fecal incontinence*

## Abstract

**Introduction:**

Pooled global prevalence of functional constipation is 14.4% based on Rome IV criteria. The condition often arises from voluntary withholding of faeces due to fear of painful defecation leading to symptoms such as infrequent and painful bowel movements, faecal incontinence, retentive posturing, and urinary complications. The data on functional constipation from developing nations like Nepal remain scarce. This study aims to find out sociodemographic and clinical characteristics of functional constipation among Nepalese children thereby facilitating early diagnosis and intervention strategies to mitigate its impact.

**Methods:**

A descriptive cross-sectional study was carried out over a six-month period, from 1st July to 30th December, 2023, at a tertiary-level hospital with sample size of 241. Children aged 1-15 years meeting the Rome IV diagnostic criteria for functional constipation were included in the study. Demographic information along with clinical history was collected and analysed using STATA13.

**Results:**

Hard stools or painful bowel movements were reported in 197 (81.74%) children, stool-withholding behavior in 98 (40.66%), and fecal incontinence in 37 (16.18%). Other associated symptoms included abdominal pain in 161 (66.79%), decreased appetite in 95 (39.00%), and gastroesophageal reflux in 83 (34.44%) cases. Rectal bleeding was observed in 51 (21.16%) and urinary incontinence in 26 (10.91%) children. A family history of constipation was noted in 71 (29.46%) cases.

**Conclusions:**

Functional constipation is a prevalent paediatric gastrointestinal disorder, significantly impacting a child’s quality of life. Early recognition and intervention are crucial to prevent complications and enhance outcomes.

## INTRODUCTION

Constipation is a common problem in children, with approximately 95% having no organic cause, known as functional constipation (FC).^1,2^ A pooled global prevalence of FC diagnosed clinically based on the Rome IV criteria is 14.4%.^3^ It occurs due to repeated attempts of voluntary withholding of feces by a child who tries to avoid unpleasant defecation because of fears associated with evacuation.^4^

Functional constipation is more prevalent in pre-schoolers and has been associated with positive family history, formula feeding, cow milk protein allergy, stress, and food habits.^5-7^ Children present with symptoms like infrequent painful defecation, fecal incontinence, abdominal pain, hard stool, bloating, retentive posturing, urinary incontinence, and urinary tract infections. ^8-10^

There are very limited published data regarding the prevalence of FC in developing countries, including Nepal. This study was designed to assess the sociodemographic and clinical characteristics of FC among the children of Nepal, which might aid in early diagnosis and intervention.

## METHODS

A descriptive cross-sectional study was conducted to determine the sociodemographic and clinical profile of FC among children in Nepal using a quantitative analytical method. Children aged one to 15 years of either gender who attended the pediatric OPD during the study period and fulfilled the Rome IV diagnostic criteria for FC were included in the study. This study was conducted in Shree Birendra Hospital, Kathmandu, Nepal. Ethical approval was taken from the Institutional Review Committee (IRC) of the Nepalese Army Institute of Health Sciences (Reference number 857), and data collection was carried out over six months, from July 1 to December 30, 2023.

The purpose of the study and information about the research were explained by the principal investigator to the parents and/or children in simple Nepali language for easy understanding. The child was included in the study after the parents’ consent. Written informed consent was taken prior to the study from the parents, and assent was obtained for children seven years and above. Children meeting the inclusion criteria were included in the study. Children already on drug therapy for constipation, on Iron therapy, children with acute illnesses and known cases of Hirschsprung disease, Inflammatory bowel disease, Celiac disease, GI tuberculosis, Cystic fibrosis, Cow’s milk protein allergy, Hypothyroidism, Developmental delay and or failure to thrive were excluded from the study.

Non-probability consecutive sampling was used. Eligible children aged 1-15 years with functional constipation who presented to the pediatric OPD during the study period and met the Rome IV diagnostic criteria were enrolled consecutively until the required sample size was achieved. The minimum sample size was calculated using Cochran’s single-proportion formula for descriptive studies, using an expected prevalence (p) of 18.2%, a 95% confidence level, and a 5% margin of error, which yielded a required sample size of 229.^11,12^ After adding 5% for anticipated non-response, the final target sample size was 241.

Measurement error was tried to be reduced by allowing parents time to respond to questions and confirming their understanding of the questions before documenting their response. Selection bias was minimized by stringently following the inclusion and exclusion criteria.

Data were collected using a questionnaire based on standard ROME IV diagnostic criteria for FC as well as a structured questionnaire adapted from relevant literature.^3^ The inclusion and exclusion criteria were explained, and interested participants were screened for eligibility. Eligible participants, after taking consent, were interviewed face to face using a structured questionnaire. Various sociodemographic and clinical data, including age, gender, body mass index (BMI), age at school enrollment, age when toilet training was commenced, and family history of constipation, were collected. Additionally, symptoms indicative of FC as per the Rome IV criteria were evaluated. Other associated factors, such as signs of gastroesophageal reflux, abdominal pain, reduced appetite, abdominal bloating, rectal bleeding, palpable fecal masses in abdominal examination, anal fissures, and urinary symptoms, were also documented in the pre-formed proforma.

The Rome IV diagnostic criteria for functional constipation include 1 month of at least 2 of the following in children up to 4 years of age: 2 or fewer defecations per week with a history of excessive stool retention, painful or hard bowel movements, large-diameter stools, presence of a large fecal mass in the rectum. In toilet-trained children, additional criteria may be used, at least 1 episode/week of incontinence after the acquisition of toileting skills, with a history of large-diameter stools that may obstruct the toilet. In children with developmental age >4 years, criteria include 1 month of at least 2 of at least once per week 2 or fewer defecations in the toilet per week along with at least 1 episode of fecal incontinence per week, history of retentive posturing or excessive volitional stool retention, painful or hard bowel movements, Presence of a large fecal mass in the rectum or a history of large-diameter stools that may obstruct the toilet. The symptoms must not be fully explained by another medical condition and must be insufficient to fulfill the diagnostic criteria of irritable bowel syndrome.

Statistical analysis was conducted in Statistical Software Package (STATA) version 13 (STATA Corporation, College Station, TX, USA). Descriptive statistics were used to describe the characteristics of the sample. Frequencies and percentages were computed for categorical variables, while means and standard deviations were computed for continuous variables.

## RESULTS

Among 241 children with functional constipation, 150 (62.24%) were in early childhood (2-5 years), followed by 62 (25.72%) toddlers aged between one and two years. A total of 160 (66.39%) children were male. The mean body mass index (BMI) of the participants was 15.5±2.9 kg/m^2^. Regarding school admission age, 162 (67.22%) children were admitted between 2-5 years, while 42 (17.43%) were admitted before 2 years of age. A family history of constipation was present in 71 (29.46%) children (Table 1).

**Table 1:** Sociodemographic traits of the children with functional constipation (n=241).

Variable	n(%)
**Age**	
Toddlers (1-2 years)	62(25.72)
Early childhood (2-5 years)	150(62.24)
Middle Childhood (6-11 years)	13(5.39)
Early Adolescents (12-15 years)	16(6.64)
**Gender**	
Male	160(66.39)
Female	81(33.61)
**Age of School admission**	
<2 years	42(17.43)
2-5 years	162(67.22)
>5 years	17(7.05)
Not started	20(8.29)
Family history of constipation	71(29.46)

**Table 2:** Symptoms and signs related to Rome IV diagnostic criteria for functional constipation (n=241).

Variable	n(%)
≤2 stool in the toilet per week	117(48.55)
Hard bowel movements with or without painful bowel movements	197(81.74)
Painful bowel movements	152(63.07)
Retentive posturing or Excessive Stool retention	98(40.66)
Faecal incontinence in toilet trained child	37(16.81)
(220 children were toilet trained)	
Large diameter stool	57(23.65)
Large faecal mass per rectum in DRE (DRE was done in 233 participants)	163(69.96)

**Table 3:** Symptoms in children with functional constipation (n=241).

Symptom	n(%)
Pain abdomen	161(66.80)
Decreased appetite	95(39.41)
Gastroesophageal reflux	83(34.44)
Rectal bleeding	51(21.16)
Urinary incontinence	26(10.79)

**Table 4:** Clinical examination findings in children with functional constipation (n=241).

Clinical Examination	n(%)
Palpable faecal mass per abdomen	55(22.82)
Distended abdomen	27(11.20)
Anal fissure	14(5.81)

There were 197 (81.74%) patients with functional constipation had hard bowel movements with or without painful bowel movements, 152 (63.07%) patients had Painful bowel movements and 117 (48.55%) patients had history of ≤2 stool in the toilet per week. Out of 241 participants, digital rectal examination (DRE) was done in 233, among which 163 (69.96%) had impacted stool in the rectum (Table 2).

Out of 241 children, 161 (66.80%) patients presented with pain abdomen, 95 (39.41%) patients presented with decreased appetite and 83 (34.44%) presented with gastroesophageal reflux (Table 3).

Among 241 participants with functionalconstipation, 55(22.82%) patients had palpable fecal massper abdomen, 27(11.20%) patients had a distended abdomen, and 14 (5.81%)patients had anal fissures (Table 4).

Out of 220 participants with functional constipation who have already attended their bladder control, 26(10.79%) had a history of urinary incontinence. None of participants gave a history of urinary retention. Regarding the initiation of toilet training among participants, 78 (32.45%) children started toilet training at 30 to 36 months of age, while 24 (10.00%) children began early toilet training before 18 months of age.

## DISCUSSION

Our study found that constipation was most prevalent in early childhood between the ages of 2 to 5 years, consistent with previous studies that support rise of prevalence among infants and toddlers.^6^ Functional constipation in children may be influenced by the timing and method of toilet training, which has been shown to affect bowel patterns. The increased prevalence of functional constipation among children aged 2 to 5 years aligns with the age range for school admission, as observed in present study. This correlation suggests that the onset of constipation symptoms may coincide with the transition into structured educational settings. A school based study done in Dhaka Division, Bangladesh revealed that inadequate toilet numbers at school increases the risk of constipation by 2.5times as compared to adequate toilet numbers(OR = 2.493, 95%CI: 1.214-5.120). Similarly children who feel embarrassed to use toilet at school had 3.6 times higher risk of constipation (OR = 3.552, 95%CI: 1.435-8.794).^7^ Further, a positive family history of constipation was observed in 29.46% children with functional constipation. This finding suggests that positive family history may have a contributory role in the development of functional constipation. Genetic predispositions could be one of the reasons though it is rarely discussed and addressed in literatures. Further research is needed to explain potential genetic influences and their role in pathophysiology of functional constipation.

In the current study, majority (32.4%) of the participants’ toilet training was initiated between 30 and 36 months of age. Only a small percentage (less than 10%) was initiated before 18 months of age. Previous research indicates that there is higher chance of developing functional constipation for both early (less than 18 months) and delayed (more than 36 months) initiation of toilet training.^8^ This could be because of behavioural resistance or lack of preparation during a crucial developmental period.^13^ Delays in toilet training can also result in prolonged stool retention, which can cause painful defecation and faecal impaction which are two major symptoms of functional constipation. Stressful or forceful toilet training is associated with stool withholding behaviours and chronic constipation. Furthermore, children who receive premature toilet training may develop anxiety regarding defecation, which leads to avoidance and worsens constipation symptoms.^8^

The majority of study participants (81.74%) reported hard stools, and only with few (2.9 %) had history of passing loose stool (29.88%). Stool retention was observed in 40.66% of respondents, which is closely in line with findings from a Korean study where 45% of participants reported similar symptoms.^14^ Furthermore, 63.1% of children with functional constipation experienced painful bowel movements. Multiple studies conducted in different regions shows that there is a significant prevalence of painful defecation among those with functional constipation which is consistent with this conclusion.^2,9,10^ Painful bowel defecation is a common clinical characteristic offunctional constipation, and the consistency of the research emphasises the importance of early diagnosis and timely intervention. Faecal incontinence, defined as a frequency of at least once per week in toilet trained child, was observed in 16.18% of children in current study. This incidence is comparatively lower than that reported in previous studies conducted across various countries, where the prevalence of faecal incontinence among children with functional constipation was found to be 18.3%.^1,11^

In our study, among children with functional constipation who had already achieved bladder control, 10.9% had a history of urinary incontinence. This study tends to show the well-documented relationship between functional constipation and urinary tract dysfunction in children. The present study aligns positively with earlier research which suggests that urinary symptoms like urgency and incontinence can be increased by constipation, which may bring about pressure on the bladder or cause dysfunctional elimination syndromes.^11^ Our study presents that 21.16% of children with functional constipation had history of rectal bleeding which is higher than that of the previous studies done in Turkey and Switzerland.^15^ Abdominal pain was found to be 66.80% which is consistent with many other existing studies like Maffei et al.^16,17^ In the study urinary tract infection is found to be 14.52% which is consistent with other relevant studies.^18^

The Rome IV criteria were applied in this study to collect details about the clinical characteristics of functional constipation among children in Nepal. The current findings suggest that the most common symptoms of functional constipation include painful defecation, hard stools, and stool retention along with pain abdomen, rectal bleeding and urinary tract infection. The clinical characteristics identified in this study align with patterns seen in other Asian nations.

The study was single centred observational study with small sample size. The result should be carefully interpreted for the general population.

Functional constipation among children in Nepal is increasingly recognized as a common issue similar to other parts of the world. However, due to false complaints, recurrences, and reluctance to consult a paediatrician and gastroenterologist, the effective management of childhood constipation has become difficult for doctors.^14^ In Nepal, research on functional constipation among children is limited. Hence this study aims to contribute valuable data on clinical characteristics of FC, thereby enhancing the understanding and management of functional constipation in the paediatric population.

## CONCLUSION

Functional constipation often manifests through symptoms such as hard stools, painful defecation, and stool withholding behaviours. These symptoms can lead to additional concerns of abdominal pain. Moreover, FC is frequently associated with urinary incontinence and urinary tract infections.

## Data Availability

The data are available from the corresponding author upon reasonable request.

## References

[1] Van den Berg MM, Benninga MA, Di Lorenzo C (2006). Epidemiology of childhood constipation: a systematic review.. Am J Gastroenterol.

[2] Loening-Baucke V (2005). Prevalence, symptoms, and outcome of constipation in infants and toddlers.. J Pediatr.

[3] Jativa-Marino E, Rivera-Valenzuela MG, Velasco-Benitez CA, Saps M (2019). The prevalence of functional constipation in children was unchanged after the Rome IV criteria halved the diagnosis period in Rome III.. Acta Paediatr.

[4] Tran DL, Sintusek P (2023). Functional constipation in children: what physicians should know.. World J Gastroenterol.

[5] Van Tilburg MAL, Hyman PE, Walker L, Rouster A, Palsson OS, Kim SM (2015). Prevalence of functional gastrointestinal disorders in infants and toddlers.. J Pediatr.

[6] Chan AOO, Lam KF, Hui WM, Leung G, Wong NYH, Lam SK (2007). Influence of positive family history on clinical characteristics of functional constipation.. Clin Gastroenterol Hepatol.

[7] Walter AW, Hovenkamp A, Devanarayana NM, Solanga R, Rajindrajith S, Benninga MA (2019). Functional constipation in infancy and early childhood: epidemiology, risk factors, and healthcare consultation.. BMC Pediatr.

[8] Van Dijk M, Benninga MA, Grootenhuis MA, Last BF (2010). Prevalence and associated clinical characteristics of behavior problems in constipated children.. Pediatrics.

[9] Yazbeck S, Schick E, O'Regan S (1987). Relevance of constipation to enuresis, urinary tract infection, and reflux.. Eur Urol.

[10] Oswari H, Alatas FS, Hegar B, Cheng W, Pramadyani A, Benninga MA (2018). Epidemiology of paediatric constipation in Indonesia and its association with exposure to stressful life events.. BMC Gastroenterol.

[11] Chaokromthong K, Sintao N (2021). Sample size estimation using Yamane and Cochran and Krejcie and Morgan and Green formulas and Cohen Statistical power analysis by G*Power and comparisons.. Apheit Int J.

[12] Russo M, Strisciuglio C, Scarpato E, Bruzzese D, Casertano M, Staiano A (2019). Functional chronic constipation: Rome III criteria versus Rome IV criteria.. J Neurogastroenterol Motil.

[13] Lacy BE, Mearin F, Chang L, Chey WD, Lembo AJ, Simren M (2016). Bowel disorders.. Gastroenterology.

[14] Chang SH, Park KY, Kang SK, Kang KS, Na SY, Yang HR (2013). Prevalence, clinical characteristics, and management of functional constipation at pediatric gastroenterology clinics.. J Korean Med Sci.

[15] Dehghani SM, Kulouee N, Honar N, Imanieh MH, Haghighat M, Javaherizadeh H (2015). Clinical manifestations among children with chronic functional constipation.. Middle East J Dig Dis.

[16] Gijsbers CF, Kneepkens CF, Vergouwe Y, Buller HA (2014). Occult constipation: faecal retention as a cause of recurrent abdominal pain in children.. Eur J Pediatr.

[17] Maffei HV, Moreira FL, Kissimoto M, Chaves SM, Faro SE, Aleixo AM (1994). Clinical and alimentary history of children attending a pediatric gastroenterology outpatient clinic with functional chronic constipation and its possible complications.. J Pediatr (Rio J).

[18] Loening-Baucke V (1997). Urinary incontinence and urinary tract infection and their resolution with treatment of chronic constipation of childhood.. Pediatrics.

